# Social support enhances the mediating effect of psychological resilience on the relationship between life satisfaction and depressive symptom severity

**DOI:** 10.1038/s41598-023-31863-7

**Published:** 2023-03-24

**Authors:** Yun-Hsuan Chang, Cheng-Ta Yang, Shulan Hsieh

**Affiliations:** 1 Institute of Gerontology, College of Medicine, National Cheng Kung University, No. 1, University Road, Tainan City, 701 Taiwan, ROC; 2 Institute of Behavioral Medicine, College of Medicine, National Cheng Kung University, Tainan City, 701 Taiwan, ROC; 3 Department of Psychology, National Cheng Kung University, No.1, University Road, Tainan City, 701 Taiwan, ROC; 4 Institute of Genomics and Bioinformatics, College of Life Sciences, National Chung Hsing University, Taichung City, 402 Taiwan, ROC; 5 Graduate Institute of Mind, Brain and Consciousness, Taipei Medical University, Taipei, 110 Taiwan, ROC; 6 Institute of Allied Health Sciences National Cheng Kung University, Tainan City, 701 Taiwan, ROC; 7 Department of Public Health, National Cheng Kung University, Tainan City, 701 Taiwan, ROC

**Keywords:** Psychology, Health care

## Abstract

Psychosocial factors, including life satisfaction, resilience, and social support, have been proposed to influence depressive symptom severity in adults because the age of onset of depressive disorders, i.e. adolescence to early adulthood, is associated with various impairments in psychosocial functioning. In this study, a psychosocial model was constructed to verify these relationships to prevent depression. For this study, 370 participants were recruited from the community via poster or online advertisements. They completed several questionnaires to assess depressive symptom severity: the Connor-Davidson Resilience Scale (CD-RISC), Satisfaction with Life Scale (SwLS), Peace of Mind (PoM) scale, Social Support Questionnaire (SSQ), and Beck Depression Inventory (BDI-II). A negative association was found between depressive symptom severity and all other variables, including PoM and CD-RISC scores, life satisfaction, and social support. Such factors can be considered protective against increased depressive symptom severity. In addition, indirect effects of PoM and resilience on the negative association between SwLS scores and depressive symptom severity were observed. Moreover, social support was found to mediate the correlation between PoM and resilience, implying that social support mediates the relationship between state of mind and resilience. The psychosocial model suggested that depressive symptom severity is influenced by internal factors (an individual’s state of mind, subjective view of events and their life) and external factors (including social support).

## Introduction

Depression has been identified as the leading cause of disability and the second most prevalent mental illness in 2020, according to the Global Burden of Disease^1^. Being mentally ill can reduce work performance and social functioning and poses a significant economic burden to society if left untreated. In addition, depression is associated with various psychosocial dysfunctions^2^. In the last 2 years, the world has been affected by the COVID-19 pandemic, which has caused uncertainty and unpredictability in the social, economic, and international spheres as well as living conditions^3,4^. Public health policies in response to the pandemic have restricted people’s lives, as they have had to adhere to quarantine and lockdown requirements and maintain social distancing. This extremely stressful situation led to decreased self-efficacy and autonomy, inducing negative emotions, such as anxiety and depression^4,5^. Such instability and reduced autonomy, which reduce life satisfaction, have greatly impacted mental health^6^. The implementation of mandatory policies to reduce the spread of the virus have impacted psychological well-being and mental health^5,7,8^. A study using an online survey for healthcare workers during the COVID-19 pandemic showed that resilience and perceived social support could prevent negative impacts on mental health among healthcare workers^9^.

In addition, the fear of catching such an infectious disease can also affect life satisfaction^10,11^. Life satisfaction, a subjective and cognitive evaluation of one’s life according to one’s goals and achievements^10^, has been described as one dimension of mental health^12^ and an important component of quality of life^13^. Lower satisfaction with life (SwL) has been associated with an elevated risk of mental illness, such as depressive disorders. Moreover, the level of life satisfaction has been identified as a predictor for depressive symptoms in a long years later^14,15^. Thus, level of life satisfaction has gained attention because of its positive association with positive mental health, including longevity^16^, and being harmonious and happy^17^. Recent research has reported that satisfaction with life can be meaningfully attained through harmony in life^18,19^. The concept of life satisfaction includes making evaluations and comparisons between actual and expected life circumstances^18^.

The factors related to life satisfaction include one’s concept and evaluation and can thus be measured with subjective well-being^20,21^. Subjective well-being refers to an individual’s experience and evaluation of specific domains and activities in their lives in terms of life satisfaction. Cultural factors play an important role in subjective well-being and mental health under Asian perceptions of health, derived from the teaching and philosophies of Confucianism^22^. Peace of mind (PoM) has been identified as a factor influencing an individual’s mental state and is correlated with one’s feelings and evaluation of overall mental state^23^. In addition, harmony can be defined as a state of balance within an individual’s mind that emphasizes self-control and emotional regulation with the surroundings to cultivate a socially conscious self^24^. Maintaining a harmonious and happy mental state (affective well-being) is valued in Chinese culture and is referred to as PoM^25^. PoM is described as a stable emotional state that is indicative of inner stability and spiritual of an ideal positive state with increased sensitivity to the outside environment. The Chinese concept of well-being refers to “a dynamic process of achieving and maintaining a good fit from within and outward”^24^; thus, PoM likely improves well-being and decreases the risk of mental illness.

A strong sense of PoM sometimes accompanies a high level of spirituality and calmness while facing life’s challenges, and positive associations of PoM with quality of life as well as resilience have been suggested^26,27^. Psychological resilience is measured when facing diverse challenges, threats, and stresses^28^ and refers to the dynamic process of adapting to these diverse stressors and recovering from adverse experiences. Moreover, based on previous empirical studies, resilience is negatively correlated with mental illness, such as depression^28–30^, and positively associated with indicators of mental health, such as life satisfaction and social support^31^.

According to Cohen’s^31^ definition, social support refers to the psychological and physical resources provided by social networks that help individuals cope with stress. In addition, social support has also been defined as accessible support for an individual from other individuals or groups^32,33^, and has been identified as crucial to the theoretical and causal impact of social relationships on health^34^. Kaufman et al.^35^ addressed the important role of social support in conferring resilience to stress by examining its moderating effects on the relationship between genetic risks for depression and depression in maltreated children. Social support-related factors have been suggested to help maintain mental health and reduce morbidity and mortality from medical illness; social support can enhance an individual’s resilience when facing stress^36^. Social support was found to mediate the relationship between pandemic stress and risks of depression and anxiety in Korean young adults^7^. In addition, for individuals suffering from lung cancer^37^ or exposure to trauma^38^, social support was found to mediate the relationship between psychological resilience and depression or anxiety. Moreover, a study in medical workers, a chain mediation effect of social support in between the psychological resilience and depression affecting life satisfaction was reported^39^. In other words, social support can be a subjective or objective factor; subjective social support was partially mediated by psychological resilience, while objective social support was completely mediated by psychological resilience and anxiety^37^.

During stressful situations (i.e. the COVID-19 pandemic, cancer or trauma), the role of psychological resilience in the relationships of social support, mental state, and life satisfaction with depressive symptom severity remains unclear. This study constructed a hierarchical psychosocial model to examine depressive symptom severity. In this hierarchical model, social support mediated psychological resilience with PoM reducing the risk of depression.

### Aim and objectives

The current study aimed to identify associations among psychosocial factors related to mental health among adults. The specific objectives were as follows: (1) to identify whether psychological resilience mediates the relationship between life satisfaction and depressive symptom severity; (2) to identify whether PoM and psychological resilience mediate the relationship between life satisfaction and depressive symptom severity; and (3) to evaluate the suitability of the model using structural equation modeling (SEM).

## Materials and methods

The study was approved by the Research Ethics Committee of National Cheng Kung University (NCKU No. 109-419) and the Institute of Review Board (IRB, JA-109-95) of Jen-Ai Hospital. All participants were given a full explanation about the study and signed an informed consent form before participating in the research. This study was performed in accordance with the principles of the Declaration of Helsinki. In addition, all participants reimbursed for transportation costs. Data were anonymized and collected in confidentiality.

### Participants

Participants without a history of neurological or psychiatric disorders or cardiovascular diseases (as self-reported) were recruited via online and poster advertisements and instructed to complete questionnaires. According to MacCallum et al.’s estimation of root mean square error of approximation (RMSEA), a sample size of at least 200 was suggested^40^. In addition, Schumacker and Lomax^41^ reviewed studies and reported that in most studies, most sample sizes consisted of 205–500 individuals. They further noticed that in studies with a small sample size (of 100–150), severe estimation problems may occur, such as failure to converge and unacceptable solutions^42^. In the current study, we used G*Power to estimate the sample size, using an effect size of 0.1, an α of 0.05, a power of 95%, and 9 predicted variables. An estimated sample size of 245 participants was calculated. Each participant was instructed to complete the following questionnaires: the Chinese versions with traditional Chinese characters of the Social Support Questionnaire (SSQ), the Satisfaction with Life Scale (SwLS), the Beck Depression Inventory-II (BDI-II), the Peace of Mind (PoM) scale, and the Connor–Davidson Resilience Scale (CD-RISC).

### Instruments

#### Depressive symptom severity

The Chinese version of the Beck Depression Inventory-II (BDI-II) is applied to evaluate the severity of depressive symptoms in normal and psychiatric populations^43,44^. The original BD-II is a 21-item inventory used to evaluate people’s (self-reported) feelings in the last 7 days. Each item is scored from 0 to 3. A higher score indicates more severe depression (< 10, normal; 10–18, mild; 19–29 moderate; 30–63, severe)^45^. The Chinese version of the BPI-II showed good reliability, with a Cronbach’s α of 0.87^46^. The Cronbach’s α of the BDI-II in the current study was 0.90.

#### Connor-davidson resilience scale (CD-RISC)

The original CD-RISC is a 25-item resilience instrument with five domains: personal competence (CD-RISC_pc), trust in one’s instincts (CD-RISC_tru), positive acceptance of change (CD-RISC_acc), control (CD-RISC_con), and spiritual influences (CD-RISC_spi); good psychometric and test–retest reliability are shown by Cronbach’s α values of 0.89 and 0.87, respectively^47^. E Each item is rated on a 5-point Likert scale (0–4), with higher scores indicating greater resilience. The original version was later translated into traditional Chinese characters and modified, showing good reliability with Cronbach’s α of 0.953 and test–retest reliability of 0.798^48,49^. The Cronbach’s α of the CD-RISC in the current study was 0.923.

#### Social support questionnaire (SSQ)

The original 27-item SSQ was developed to evaluate perceived social support [in terms of the number of individuals providing social support; SSQ(N)] and satisfaction with the support [SSQ(S)]^50^. A Chinese version was first developed by Wu^51^, and shortened and modified to 20 items by Chang^52^. The internal reliability of the SSQ(N) was 0.91 and that of the SSQ(S) was 0.92 in Chang’s study. The 2-week test–retest reliability of the SSQ(N) and SSQ(S) was 0.9269 and 0.9369, respectively. In the current study, we had similar internal reliability of the SSQ(N) and SSQ(S), with Cronbach’s α values of 0.932 and 0.923.

#### Satisfaction with life scale (SwLS)

The Chinese version of the Satisfaction with Life Scale (SwLS) is a 5-item questionnaire (scored from 1 = strongly disagree to 7 = strongly agree) that measures subjective well-being^10^. Cronbach’s α of this scale was 0.87, and the 2-month test–retest reliability was 0.82^53^. Several studies have reported that this scale reflects only one factor^53–55^. The Chinese version of the SwLS was defined and validated with a Cronbach’s α of 0.96^56^. The Cronbach’s α value of the SwLS in the current study was 0.923.

#### Peace of mind (PoM) scale

PoM is defined as an internal state of peacefulness and harmony. The construct was originally developed based on the description of well-being valuation in Chinese culture^25^. A cross-cultural validation of this scale was conducted and showed cultural differences, with a higher PoM score for Taiwanese individuals than for Europeans or Americans. The Cronbach’s α of PoM in the current study was 0.610.

### Data analysis

Pearson’s correlation analysis was conducted to explore the associations among variables. SPSS 22.0 (SPSS Inc., Chicago, IL, USA) was to conduct the statistical analysis. To further explore the mediating role of resilience in the association between life satisfaction and depression, the mediating role of PoM in the relationship between life satisfaction and depression, and the mediating role of social support in the relationship between PoM and resilience, bootstrap analysis was carried out. Structural equation modeling (SEM) was employed to empirically test the hypothesis that the number of social support providers indirectly mediates the relationship between well-being and resilience, in which PoM plays a double mediating role in the relationship between depression and social satisfaction. The goodness-of-fit index was computed to evaluate model fit, with RMSEA < 0.08^57,58^.

### Ethical approval and consent to participate

The study was approved by the Research Ethics Committee (REC) at National Cheng Kung University (NCKU No. 109-419) and the Institute of Review Board (IRB, JA-109-95) of Jen-Ai Hospital. All participants were given a full explanation of the study and signed an informed consent form agreeing to join the research.

## Results

### General characteristics of participants

A total of 395 participants with no self-reported mental or neurological disorders were recruited via an online platform from October 2019 to December 2021. Of these, 307 (80.43%) participants (mean age: 26.84 years, SD = 12.69 years) completed all questionnaires and were paid $500 New Taiwan Dollars (NTD). Among the participants, 168 were men, with a mean age of 27.84 ± 14.07 years, and 202 were women, with a mean age of 26.01 ± 11.41 years.

### Correlations among depression, resilience, and life satisfaction

We conducted Harman’s one-factor test to detect common method bias. The results showed that the cumulative variance explained was 22.728%, which is lower than 50%. Thus, no significant common method bias was detected in the current study according to Podsakoff et al.’s suggestion^59^.

The scores on each scale are shown in Table 1. Pearson’s correlation analysis showed significant correlations among depressive symptom severity, total CD-RISC score, PoM score, and SwLS score (*ps* < *0.05*). Significant negative correlations were found between depressive symptom severity and CD-RISC and PoM scores, and significant positive associations were found between CD-RISC scores and PoM and SwLS scores (*ps* < *0.05*).

**Table 1:** Mean and standard deviation (SD) of scores on the questionnaires.

Scale or subscale		Mean	SD
SwLS score	SwLS	22.04	6.78
BDI-II total score	BDI-II	7.76	7.10
PoM score	PoM	3.46	0.77
CD-RISC_t score	CD-RISC_t	65.06	15.16
CD-RISC_pc score	CD-RISC_pc	20.71	5.89
CD-RISC_tru score	CD-RISC_tru	18.30	4.21
CD-RISC_acc score	CD-RISC_acc	13.57	3.33
CD-RISC_con score	CD-RISC_con	7.39	2.43
CD-RISC_spi score	CD-RISC_spi	5.09	1.59
SSQ(N)	SSQ(N)	25.65	15.68

*SwLS* satisfaction with life scale, *BDI-II* beck depression inventory-II, *CD-RISC* Connor-Davidson resilience scale, *CD-RISC_t* CD-RISC total, *CD-RISC_pc* CD-RISC personal competence, *CD-RISC_tru* CD-RISC trust in one’s instincts, *CD-RISC_acc* CD-RISC positive acceptance of change, *CD-RISC_con* CD-RISC self-control, *CD-RISC_spi* CD-RISC spiritual influences, *SSQ(N)* number of individuals providing social support on the social support questionnaire.

### A psychosocial model of resilience and social support

**Figure 1:**
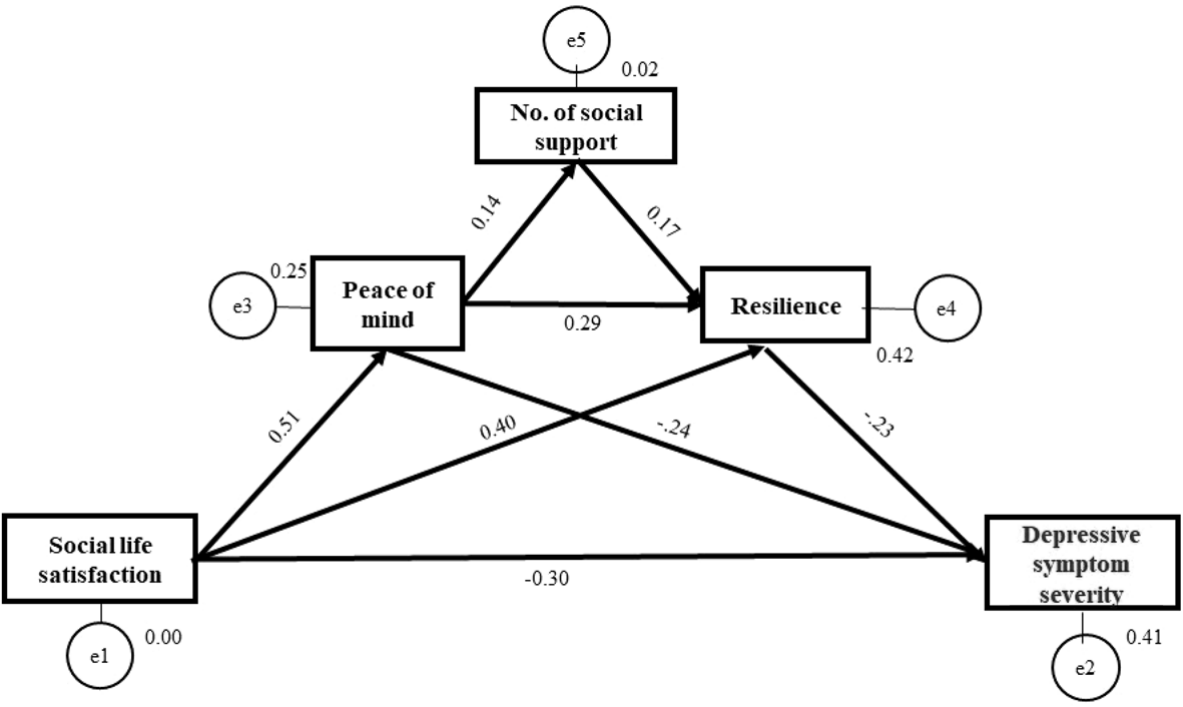
A hierarchical psychosocial model of depressive symptom severity in adults.

The hypothesized psychosocial model of social support, PoM, resilience, and depressive symptom severity was constructed using SEM. The dimensions of resilience in the CD-RISC were included in the model as latent variables, and the unidimensional constructs of depression and life satisfaction were included as observable variables. The goodness-of-fit indices of the model (Fig. 1) indicated good fit to the data, and the model was acceptable (χ^2^/df = 1.399, p = 0.247; RMSEA = 0.033, standardized root mean squared residual [SRMR] = 0.0174, comparative fit index [CFI] = 0.998, incremental fit index [IFI] = 0.998).

Effect analysis using bootstrapping was applied to identify the direct, indirect, and total effects on depressive symptom severity; the results are shown in Table 2. Life satisfaction had significant negative direct (*β* = − 0.301; *p* = 0.001), indirect (*β* =  − 0.247, *p* = 0.001), and total (*β* = − 0.549; *p* = 0.001) effects on depressive symptom severity. Resilience (*β* = − 0.228; *p* = 0.001) had a significant direct effect on depressive symptom severity, while PoM had significant direct and indirect effects on depressive symptom severity (*β* = − 0.240; *p* = 0.001 and *β* = − 0.072, *p* < 0.0005, respectively). The number of social support providers had a significant indirect effect on depression (*β* = − 0.038, *p* < 0.0005) and a significant direct effect on resilience (*β* = 0.166, *p* = 0.001). PoM had a significant direct effect (*β* = 0.139, *p* = 0.005) on the number of social support providers.

**Table 2:** Standardized estimates of total, direct, and indirect effects of variables on depression and mediator variables.

		Total effect		Direct effect		Indirect effect	
		*β*	*p*	*β*	*p*	*β*	*p*
Depressive symptom severity	←*SwLS score*	− 0.549	0.001	− 0.301	0.001	− 0.247	0.001
	←CD-RISC score	− 0.228	0.001	− 0.228	0.001	–	–
	←*PoM score*	− 0.312	0.002	− 0.240	0.002	− 0.072	< 0.0005
	←*SSQ(N)*	− 0.038	< 0.0005	–	–	− 0.038	< 0.0005
CD-RISC score	←*SwLS score*	0.561	0.001	0.404	0.001	0.157	0.001
	←PoM score	0.315	0.001	0.292	0.001	0.023	0.002
	←SSQ(N)	0.166	0.001	0.166	0.001	–	–
PoM score	←*SwLS score*	0.498	< 0.0005	0.498	< 0.0005	–	–
SSQ(N)	←*SwLS score*	0.069	0.004	–	–	0.069	0.004
	←PoM score	0.139	0.005	0.139	0.005	–	–

## Discussion

The findings of the current study support our hypothesis that the psychosocial model of resilience and depressive symptom severity is hierarchical. From the biological perspective of resilience, reports have revealed the importance of an individual’s mental status. People who are more optimistic and hopeful while facing extreme or chronic stress may have a reward system that becomes hypersensitive or resistant to change^60^. With such “resilience”, the reward system could help individuals to maintain an appropriate mood during highly stressful and challenging situations and reduce depressive symptom severity. Such a reward system might develop based on the sensitivity of dopamine receptors and correlate with resistance to stress-induced cerebral dopamine depletion. Highly resilient people may also exhibit high cognitive function by remaining positive and hopeful about the future while facing long periods of extreme stress. In such circumstances, psychological resilience indirectly mediates the relationship between life satisfaction and depressive symptom severity. Another model, the affective neuroscience model, indicates that psychological resilience can be boosted in adults by increasing social support, influencing resilience through several pathways^61^. Moreover, social support has been also reported its impact on reducing depressive symptoms and suicidal ideation among undergraduates during the COVID-19 lockdown^4^. The present findings are in line with previous reports on the possible enhancement of resilience by social support, particularly the number of support providers when individuals face stress and seek further support. Moreover, in young adults^6^ and elderly individuals, social support has a significant indirect effect on geriatric depression through psychological resilience^61^. Previous studies have reported correlations among social support, psychological resilience and health in Asian cultures^7,22,62–64^ (i.e. South Korea, Japan and Singapore), which implies the importance of social support in the non-Western cultures^65^. Moreover, the current finding implies that the number of social support providers plays an important role in mediating the relationship between mental state and resilience, implying the importance of social support in mental health^66,67^.

Regarding the Chinese concept linking harmony and happiness, PoM also played an important mediating role in the relationship between life satisfaction and resilience; in other words, an internal state of peacefulness and harmony mediated the relationship between life satisfaction or well-being and resilience. In addition, PoM may indirectly affect the negative association between life satisfaction and depressive symptom severity. Increased PoM may increase happiness, sense of meaning, involvement, and life satistisfaction^68^, and may enhance resilience in the face of chronic stress, decreasing symptoms of depression^69,70^.

In the last few years, the world has been affected by the COVID-19 pandemic. People experienced fear and distraction while unexpected viral strains quickly spread across the globe. Researchers have suggested that during extremely stressful circumstances, an individual’s prior social support has a time-lagged effect on PoM^71^. Previous findings support our observations, and the number of social support providers indirectly enhances an individual’s PoM and resilience.

The current study constructed a potential model to explain the relationship between social support and depressive symptom severity in terms of psychological resilience. In addition, mental state and harmony indirectly mediate the association between life satisfaction and depressive symptom severity as well as between life satisfaction and psychological resilience. PoM is a cultural factor that describes a calm and stable mental state marked by self-control and spiritual discipline and is consistent with subjective well-being in Chinese or Asian cultures^71^. Most people can adjust their emotional state; increased social support can help improve life satisfaction by meeting basic needs, stabilizing emotions, and reducing negative emotions, improving psychological resilience and coping strategies.

### Limitations and prospects

Although our findings provide an empirical and theoretical contribution to the field, there are some limitations that need to be addressed in the future. First, although the psychosocial model tested in the current study considered interactions among factors related to mental health, the study design was cross-sectional, which did not allow us to determine the causality of relationships among these factors. In addition, most participants were young adults who may not have had previous experience with extreme stress. Future studies should recruit a more homogeneous group of participants and conduct longitudinal investigations using relevant models. Second, although this study was conducted during the COVID-19 pandemic, the disease was under control in Taiwan during most of the study period; thus, individuals may not have been as affected as those in other countries. Perceptions of stress could be explored in a future study comparing the study population with other populations. Moreover, because of the long-time span of the collected data, social environment, and personal factors, the mental health problems of the participants may have a great impact and change through such complex model^72^. Third, this study showed that the number of social support providers had an indirect effect on the relationship between PoM and resilience. Future studies should consider other factors related to social support, such as the resources provided, the intimacy of providers, and content of social support, in the resilient dynamic process^73^.

## Data Availability

All data generated or analyzed during this study are included in this manuscript.
